# Multiple-model machine learning identifies potential functional genes in dilated cardiomyopathy

**DOI:** 10.3389/fcvm.2022.1044443

**Published:** 2023-01-11

**Authors:** Lin Zhang, Yexiang Lin, Kaiyue Wang, Lifeng Han, Xue Zhang, Xiumei Gao, Zheng Li, Houliang Zhang, Jiashun Zhou, Heshui Yu, Xuebin Fu

**Affiliations:** ^1^State Key Laboratory of Component-Based Chinese Medicine, Tianjin University of Traditional Chinese Medicine, Tianjin, China; ^2^Biomedical Engineering, Imperial College London, London, United Kingdom; ^3^Tianjin Jinghai District Hospital, Tianjin, China; ^4^Department of Cardiovascular-Thoracic Surgery, Northwestern University Feinberg School of Medicine, Chicago, IL, United States; ^5^Department of Pediatrics, Ann & Robert H. Lurie Children’s Hospital of Chicago, Chicago, IL, United States

**Keywords:** diagnosis value, dilated cardiomyopathy, machine learning, *SERPINA3*, *FRZB*, *FCN3*

## Abstract

**Introduction:**

Machine learning (ML) has gained intensive popularity in various fields, such as disease diagnosis in healthcare. However, it has limitation for single algorithm to explore the diagnosing value of dilated cardiomyopathy (DCM). We aim to develop a novel overall normalized sum weight of multiple-model MLs to assess the diagnosing value in DCM.

**Methods:**

Gene expression data were selected from previously published databases (six sets of eligible microarrays, 386 samples) with eligible criteria. Two sets of microarrays were used as training; the others were studied in the testing sets (ratio 5:1). Totally, we identified 20 differently expressed genes (DEGs) between DCM and control individuals (7 upregulated and 13 down-regulated).

**Results:**

We developed six classification ML methods to identify potential candidate genes based on their overall weights. Three genes, serine proteinase inhibitor A3 (*SERPINA3*), frizzled-related proteins (FRPs) 3 (*FRZB*), and ficolin 3 (*FCN3*) were finally identified as the receiver operating characteristic (ROC). Interestingly, we found all three genes correlated considerably with plasma cells. Importantly, not only in training sets but also testing sets, the areas under the curve (AUCs) for *SERPINA3*, *FRZB*, and *FCN3* were greater than 0.88. The ROC of *SERPINA3* was significantly high (0.940 in training and 0.918 in testing sets), indicating it is a potentially functional gene in DCM. Especially, the plasma levels in DCM patients of SERPINA3, FCN, and FRZB were significant compared with healthy control.

**Discussion:**

SERPINA3, FRZB, and FCN3 might be potential diagnosis targets for DCM, Further verification work could be implemented.

## 1. Introduction

Machine learning (ML), composed of various intricate algorithms, is recently commonly applied to explore potential biomarkers (e.g., lipidome, metabolome, and transcriptome) and prognosis (1, 2), especially in variable filtration (3–5). For example, MLs can recognize patterns better representing the individual risk compared to classical surgical risk scores (6). ML includes various types, such as support vector machine (SVM) (7, 8), random forest (RF) (9), decision tree (DT) (10–12), and so on. Different ML has its specialty and shortcoming. For example, least absolute shrinkage and selection operator (LASSO) processed a precision matrix of Gaussian variables using an ℓ1-penalty (13) until small values to zero but eliminated too many variables. For SVM, separated hyperplanes allow for correct partitioning and maximize geometric spacing but may be worse in a small sample size (14) compared with other MLs (15). Different ML algorithms possess both characteristics and limitations which cannot be ignored, especially in the choice of variables. Many researchers (16–18) only focus on single or two MLs which might ignore their potential shortcomings. In our previous research (19), five MLs show different weights even with the same genes. So just intersecting the top *N* genes may unconsciously delete some dominant genes (20–23). And ignoring the weights of genes may result in an imbalance of filtration (19, 24).

Dilated cardiomyopathy (DCM), not only the primary myocardial disease but also the dominant trigger in chronic heart failure (HF) (25), manifests clinically in systolic heart insufficiency and dilatation of the left ventricle (26, 27). Although there are already clinical diagnosis criteria for DCM, by the time the clinical diagnosis is clear, most of the patient’s underlying condition is poor (27). Though drugs (e.g., ivabradine) for HF are used to treat DCM and improve the prognosis in the short term (28), the long-term prognosis remains poor (29). Therefore, early diagnosis with identifying markers of DCM is necessary. Previous studies had indicated the diagnosis value of genes (30, 31) (e.g., TBX20 or Gab1) in DCM but with few microarrays (32), which means a small sample size and non-universality. Thus, developing a predictive model for DCM genetic diagnosis with multiple microarrays is necessary.

**FIGURE 1:**
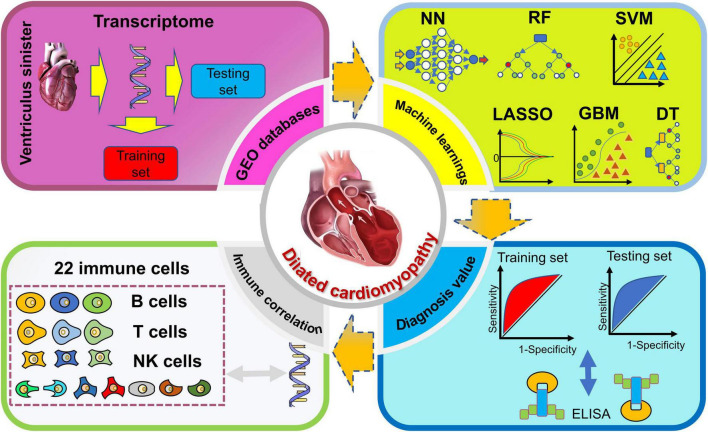
Flow chart of this study.

In this study, we identified potential transcriptomic information regarding DCM diagnosis with the overall weights in MLs of multiple microarrays. Furthermore, we further developed an immune correlation analysis between diagnosis genes and immune cells. Finally, DCM patients and healthy control were recruited for validation of related proteins of genes. The process of the following analysis (Figure 1) was shown in the flow chart.

## 2. Materials and methods

### 2.1. Data acquisition

We derived the transcriptome information of DCM from Gene Expression Omnibus (GEO). According to the following criteria, the primary data were derived with the keyword of “DCM”: (1) inclusion criteria (i) sample of the left ventricle with a diagnosis of DCM patients; (ii) transcriptome; (iii) primary data was free and accessible. (2) exclusion criteria (i) suspected carcinoma, ischemic cardiomyopathy, heart valve disease, and other diseases; (ii) intervention(s) in DCM patients.

### 2.2. Data processing

Firstly, the *sva* R package (version 3.36) was applied to eliminate branch effects and quantile normalization with the specific function of *ComBat*. Secondly, we divide all microarrays into training or testing sets with a ratio of 5:1 (33). Briefly, the training set for developing the potential diagnosis value, and the testing for verifying the results. Thirdly, we identify the differentially expressed genes (DEGs). The functional analysis of DEGs was applied through the Kyoto Encyclopedia of Genes and Genomes Gene Set Enrichment Analysis (KEGG-GSEA), Gene Ontology (GO), and Disease Ontology (DO) enrichment based on three packages, *DOSE* (version 3.22.1), *clusterProfiler* (version 4.4.4), and *enrichplot* (version 1.16.2). The GO consist of three parts, molecular function (MF), biological process (BP), and cellular components (CCs). Moreover, six MLs algorithms were applied to the classification model and filtered the candidate diagnosis genes. As for the testing group, we identify the diagnosis value of potential candidate genes. Lastly, the immune correlation between the above genes was developed.

### 2.3. Searching for DEGs

The R package, *limma* (version 3.52.4), was adopted to average the same gene expression with the function of *avereps* and then identify the DEGs. After quantile normalization, primary data sets were transformed into log2. *P*-value was adjusted to the false discovery rate based on *Benjamini and Hochberg* method. Two thresholds were set, the absolute value of fold change (|logFC|) > 1, and the false discovery rate < 0.001. With the DEGs, the heatmap and volcano plot were applied with the *pheatmap* (version 1.0.12) and *ggplot2* (version 3.3.6).

### 2.4. Classification models with six MLs

Based on the above DEGs, we further developed classification models with six MLs algorithms, SVM, LASSO, RF, gradient boosting machine (GBM), DT, and neural network (NN) to assess the classification value. Briefly, we constructed the six MLs classification models with optimized parameters in the training sets, and the testing was adopted for the validation of the six MLs. All ML models are cross-validated 10-fold to ensure stability. The accuracy value was adopted to estimate the value for six MLs and greater accuracy indicates the better classification value of the model.

The first ML (LASSO) was developed with the *glmnet* (version 4.1-4) R package. The function *cv.glmnet* was applied to optimize the value of lambda. For basic parameters, the following settings were the scale of lambda between 0 and 2,000 with one step size, the family of “binomial,” and the type measure of “class.” With the min lambda, the function *glmnet* was applied to the LASSO model in training sets with alpha (equal to 1) and a family of “binomial.”

The second ML (SVM) was adopted with *e1071* R package (version 1.7-11). The function *tune.svm* was utilized to optimize the settings parameter. For basic parameters, the following settings were the kernel of “linear,” and the cost between 1 and 20. With the best number of support vectors, the classification model was built.

The third ML (DT) was finished with two R packages, *rpart* (version 4.1.16) and *rpart.plot* (version 3.1.1). The *rpart* function was applied to the model with the method of “class,” cp value of 0.00001.

The fourth ML (RF) was adopted with *randomForest* (version 4.7-1.1). In *randomForest*, the *tuneRF* was served to optimize 500 trees and 1 step size. With the optimal trees for min error rate, the classification model of training sets was accomplished.

The fifth ML (NN) was developed with *neuralnet* R package (version 1.44.2). In *neuralnet*, the *neuralnet* was served with five layers (containing an input layer, an output layer, and three hidden layers), the *err.fct* of “sse,” and the output of linear.

The last ML, GBM, was different from the above five algorithms with more steps and prone to making. The GMB was accomplished with *h2o* (version 3.38.0.1). Only JAVA operating environment that the *h2o* can process the classification model. Thereby, we had to timely download and installed java development kit (JDK). Necessary for running memory with *h2o.init* in GBM and we adjusted the model memory of GBM to 16G. Due to the h2o data type being indispensable for GBM, we transform the data format with *as.h2o* in both the training set and testing set. Finally, *h2o.gbm* was applied to tune the parameters and model (we set the distribution of “bernoulli,” 200 trees, 0.001 for a learning rate, 0.9 for a sample rate).

Importantly, based on the above weights of six MLs for DEGs, we calculated the normalized six MLs weights of DEGs as the function in R: $O\,v\,e\,r\,a\,l\,l\,w\,e\,i\,g\,h\,t\,s=\frac{a\,b\,s\,\left(L\,A\,S\,S\,O\right)}{a\,b\,s\,\left(L\,A\,S\,S\,O\,m\,a\,x\right)}+\frac{a\,b\,s\,\left(S\,V\,M\right)}{a\,b\,s\,\left(S\,V\,M\,m\,a\,x\right)}+\frac{a\,b\,s\,\left(R\,F\right)}{a\,b\,s\,\left(R\,F\,m\,a\,x\right)}+\frac{a\,b\,s\,\left(D\,T\right)}{a\,b\,s\,\left(D\,T\,m\,a\,x\right)}+\frac{a\,b\,s\,\left(G\,B\,M\right)}{a\,b\,s\,\left(G\,B\,M\,m\,a\,x\right)}+\frac{a\,b\,s\,\left(N\,N\right)}{a\,b\,s\,\left(N\,N\,m\,a\,x\right)}$. For example, if the weight of glyceraldehyde-3-phosphate dehydrogenase (*GAPDH*) in six MLs was 15, −11, 10, −1, 160, and −4. And the max weights of absolute value in the above model were 30, 44, 40, 4, 320, and 8, respectively. The overall weight of *GAPDH* was |15| /30 + |−11| /44 + |10| /40 + |−1| /4 + |160| /320 + |−4| /8 = 2.25. Then, we filter the candidate genes for ROC (*pROC*, version 1.18.0) and immune correlation (*CIBERSORT* function) with overall weights > 1. Area under the curve (AUC) was calculated to judge the diagnosis value between control and DCM individuals.

### 2.5. Access to clinical samples

The trial complied with the Declaration of Helsinki and was approved by the Ethics Committees of the participating hospitals. All DCM patients and healthy volunteers provided written informed consent from September 20, 2022 to October 31, 2022. Ethics Committee/Institutional Review Board: Ethics Review Committee Jinghai District Hospital, Plan 11. Diary number: JHYYLL-2022-0307.

Briefly, according to the Chinese guidance (27), the inclusion criteria of DCM contain three parts, (1) left ventricular end-diastolic diameter > 5.0 cm (women) or > 5.5 cm (men); (2) left ventricular ejection fraction < 45%, left ventricular fractional shortening < 25%; (3) no other heart-related diseases and >20 years old. Blood samples were collected in ethylene diamine tetraacetic acid (EDTA)-containing tubes after a 10-h overnight fast and centrifuged at 4°C, 3,000 *g* for 10 min, then plasma was stored at −80°C. All the plasma levels of SERPINA3, FCN3, and FRZB were measured by ELISA kits (SERPINA3 Human ELISA Kit, Abcam, Cambridge, UK; Hycult Biotechnology, Uden, The Netherlands; R&D Systems, Minneapolis, MN, USA, respectively).

### 2.6. Statistical analysis

All the statistical analyses were processed by R software (version 4.1.1). CIBERSORT was adopted for immune correlation analysis. We estimate the immune correlates of 22 immune cells and visualization in the *corrplot* R package (version 0.92). For continuous variables, the independent Student’s *t*-test was adopted if the variables met Gaussian distribution, if not, the Wilcoxon test was used. A two-sided *p*-value < 0.05 was considered to be significant.

## 3. Results

### 3.1. Incorporation of microarrays

Among six microarrays (Table 1) (386 sample sizes) were finally obtained, including GSE5406, GSE57338, GSE1145, GSE1869, GSE3585, and GSE42955. According to the random ratio of 5:1, the training set was integrated with two microarrays (168 DCM and 152 healthy control), including GSE5406 and GSE57338. At the same time, the testing set was integrated with four (39 DCM and 27 control), composed of GSE1145, GSE1869, GSE3585, and GSE42955.

**TABLE 1:** Basic information on the six microarrays.

ID	Public time	Institution	Country
GSE5406	September 04, 2006	University of Pennsylvania School of Medicine	USA
GSE57338	January 01, 2015	Perelman School of Medicine at the University of Pennsylvania	USA
GSE1145	March 24, 2004	Harvard University	USA
GSE1869	October 26, 2004	Johns Hopkins Medical Institutions	USA
GSE3585	August 01, 2006	German Cancer Research Center and National Center of Tumor Diseases	Germany
GSE42955	October 17, 2013	Health Research Institute of the Hospital La Fe	Spain

### 3.2. Searching for DEGs

**FIGURE 2:**
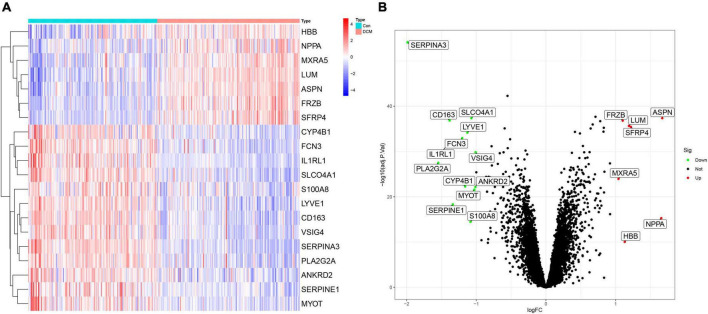
The heatmap and volcano plot of 20 differently expressed genes (DEGs) in dilated cardiomyopathy (DCM) and healthy. **(A)** The heatmap of 20 DEGs; **(B)** the volcano plot of 20 DEGs.

Among 20 DEGs with biological significance (Supplementary Table 1) from 12,937 RNAs were identified in the training sets. Compared to the healthy control, 13 genes down-regulated (SERPINA3, PLA2G2A, IL1RL1, CD163, SERPINE1, FCN3, CYP4B1, LYVE1, S100A8, SLCO4A1, MYOT, ANKRD2, and VSIG4) and 7 genes up-regulated (MXRA5, FRZB, HBB, LUM, SFRP4, NPPA, and ASPN) in the DCM individuals (Figure 2).

### 3.3. Functional enrichment analysis

**FIGURE 3:**
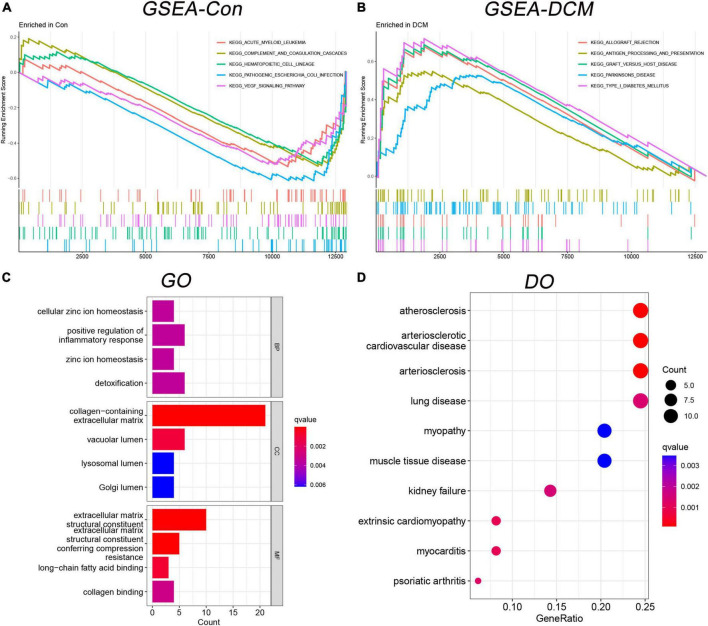
Functional enrichment analysis in gene set enrichment analysis-kyoto encyclopedia of genes and genomes (GSEA-KEGG), gene ontology (GO), and disease ontology (DO). **(A)** The GSEA-KEGG enrichment in control; **(B)** the GSEA-KEGG enrichment in DCM; **(C)** the GO enrichment term; and **(D)** the DO enrichment term.

Based on the above DEGs, we identified 21 GSEA terms (Supplementary Table 2) and show the top 5 (Figures 3A, B), 102 GO terms (Supplementary Table 3) and show the top 4 (Figure 3C), 68 DO terms (Supplementary Table 4) and show the top 10 (Figure 3D). Among GSEA-KEGG enrichments, the top 3 presented significance in Type I diabetes mellitus, graft versus host disease, and allograft rejection. Regarding the GO terms in BP, the top 3 presented significant enrichments in the cellular zinc ion homeostasis, positive regulation of inflammatory response, and zinc ion homeostasis. In terms of DO, the top 3 diseases presented were atherosclerosis, arteriosclerotic cardiovascular disease, and arteriosclerosis.

### 3.4. Six MLs algorithms for classification model and candidate genes

**FIGURE 4:**
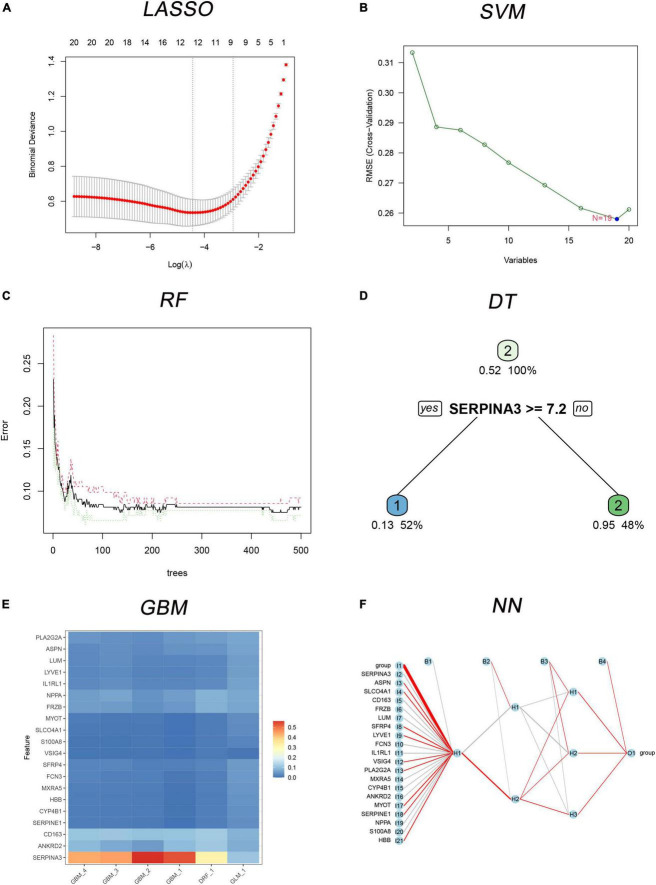
The six MLs classification models built with 20 differently expressed genes (DEGs). **(A)** Least absolute shrinkage and selection operator (LASSO) for 9 candidate genes; **(B)** support vector machine (SVM) for 19 candidates DEGs; **(C)** the error rate of the random forest (RF) classification model with increasing trees; **(D)** the decision tree (DT) for classification of control and dilated cardiomyopathy (DCM) individuals; **(E)** multiple gradient boosting machine (GBM) classification models of control and DCM individuals; **(F)** neural network (NN) for classification of control and DCM individuals.

Six classification models of MLs were successfully established (Figure 4), and we calculated the accuracy (Table 2) of both training sets and testing sets. In LASSO (Figure 4A), we filtered nine candidate genes. Disappointed, LASSO’s accuracy of the two sets were only 52.5 and 59.09%. In SVM, 19 genes were identified (Figure 4B), and the accuracies of the two sets were unstable, 90.94 and 51.52%. In RF (Figure 4C), the error rate of the classification model decreases as the number of trees increases, until 234 trees the error rate is minimized and smoothed. Surprisingly, the accuracy of the two sets was 100%. In DT (Figure 4D), thresholds of 7.2 in SERPINA3 can discriminate the health and DCM, but the accuracies of the two sets were also unstable like SVM, 93.75 and 53.03%. In GBM (Figure 4E), we developed six folds models to explore the candidate genes, but the accuracies of the two sets were also unstable, 96.03 and 53.03%. In NN (Figure 4F), enough in three hidden layers to discriminate the health and DCM, and the accuracies of both sets were 100%. Among all those models, the most important genes with the primary weights were identified (Supplementary Table 5). In the six MLs, both the RF and NN show the optimal and stable classification value. The accuracy of both MLs was 100%. Furthermore, the summation (Table 3) of normalized weights (dividing the absolute value by max weights) was calculated to screen the diagnosis genes. And nine genes (*SERPINA3*, *CD163*, *FCN3*, *LYVE1*, *SLCO4A1*, *LUM*, *FRZB*, *PLA2G2A*, and *SFRP4*) talent showing themselves with overall weights > 1 (Table 3).

**TABLE 2:** The accuracy of six classification machine learnings (MLs) in the training and testing sets.

MLs	Training set (%)	Testing set (%)
SVM	90.94	51.52
LASSO	52.5	59.09
RF	100	100
NN	100	100
GBM	96.03	53.03
DT	93.75	53.03

**TABLE 3:** The summed normalized weights of 20 differently expressed genes (DEGs) in six classifications machine learnings (MLs).

Genes	LASSO	RF	NN	GBM	DT	SVM	Sum (weights)
SERPINA3	1	1	0.73	1	1	1	5.73
CD163	0	0.37	1	0.19	0.81	0.08	2.45
FCN3	0	0.32	0.91	0.01	0.73	0.08	2.05
LYVE1	0.07	0.41	0.51	0.03	0.72	0.16	1.91
SLCO4A1	0	0.41	0.22	0.14	0.77	0.08	1.61
LUM	0.31	0.28	0.65	0.02	0	0.07	1.33
FRZB	0.18	0.35	0.25	0.09	0	0.27	1.13
PLA2G2A	0	0.21	0.1	0.02	0.73	0.04	1.09
SFRP4	0	0.15	0.83	0.02	0	0.06	1.06
NPPA	0.11	0.18	0.36	0.06	0	0.1	0.8
MYOT	0	0.11	0.64	0.01	0	0.03	0.79
ASPN	0.09	0.27	0.27	0.02	0	0.08	0.74
ANKRD2	0.27	0.18	0.09	0.07	0	0.1	0.7
MXRA5	0	0.07	0.46	0	0	0.02	0.55
HBB	0.1	0.11	0.3	0.02	0	0.03	0.55
IL1RL1	0	0.29	0.08	0.01	0	0.07	0.46
S100A8	0	0.07	0.34	0	0	0.04	0.46
CYP4B1	0.06	0.16	0.11	0.02	0	0.05	0.39
VSIG4	0	0.16	0.05	0	0	0.02	0.24
SERPINE1	0	0.08	0.08	0	0	0.02	0.18

**FIGURE 5:**
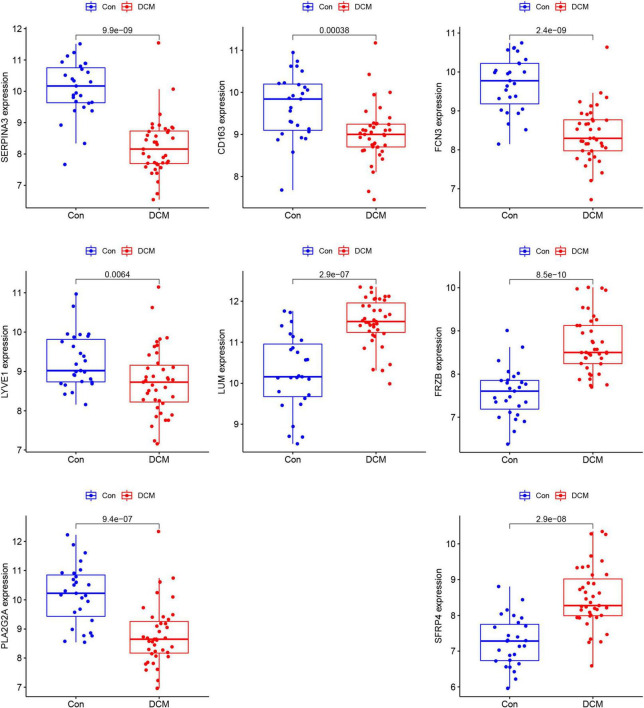
The comparison of the 8 genes between dilated cardiomyopathy (DCM) and healthy in testing sets.

Based on the summed normalized weights > 1, nine candidates genes were chosen for diagnosis in DCM and healthy individuals. Next, we validate the nine candidate genes in the testing set, and except for *SLCO4A1*, the other eight show significance (Figure 5).

### 3.5. Evaluation of the diagnosis value

**FIGURE 6:**
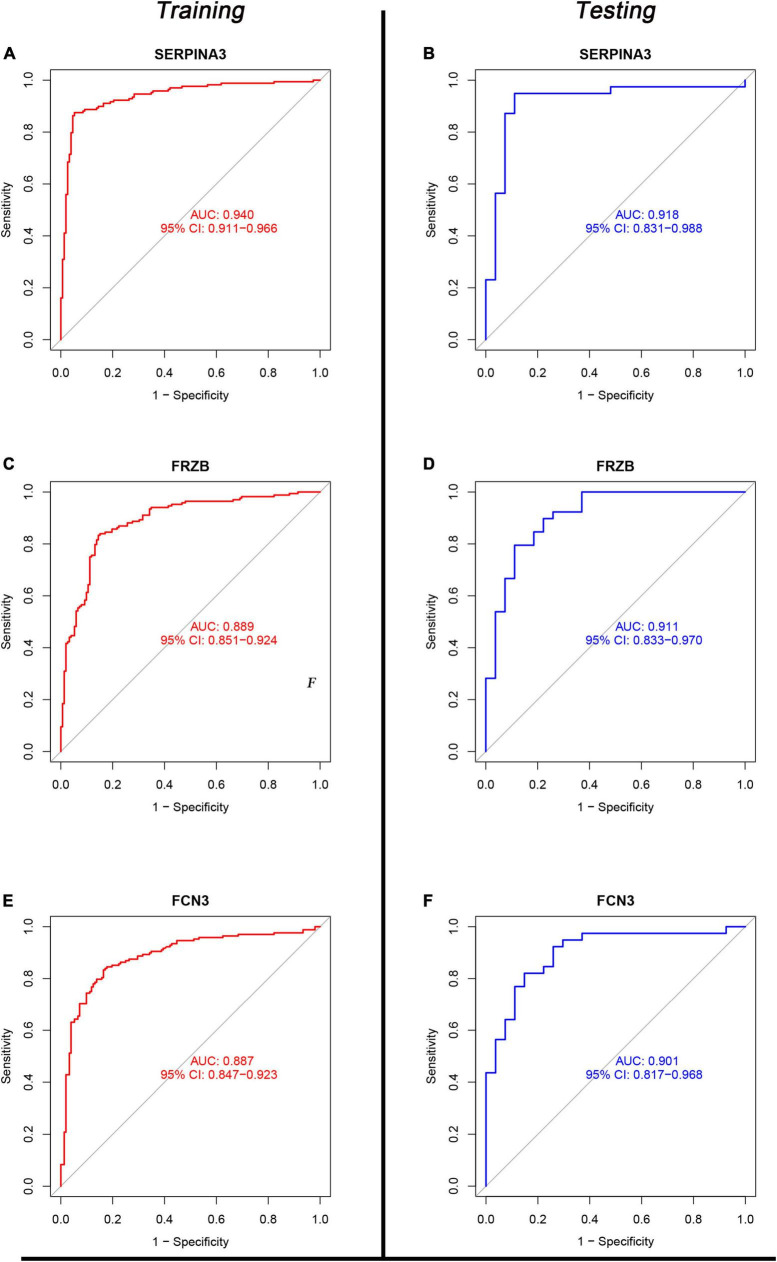
The receiver operating characteristic (ROC) of SERPINA3, FRZB, and FCN3 between the control and dilated cardiomyopathy (DCM) groups. **(A,C,E)** The ROC of SERPINA3, FRZB, and FCN3 in the Training set; **(B,D,F)** the ROC of SERPINA3, FRZB, and FCN3 in the testing set.

Eight genes (just mentioned above) were taken into the ROC curve (Supplementary Figures 1, 2). AUC values of *SERPINA3*, *FCN3*, *LUM*, *FRZB*, *PLA2G2A*, and *SFRP4* were higher than 0.8 in both two sets. Moreover, three genes *SERPINA3*, *FCN3*, and *FRZB* were higher than 0.88 (Figure 6) in the training sets and even >0.9 in the testing sets. Especially, *SERPINA3* was higher than 0.9 in both sets. In a word, three genes, *SERPINA3*, *FCN3*, and *FRZB* may be the potential diagnosis genes compared with DCM and healthy control.

### 3.6. Immune correlation

**FIGURE 7:**
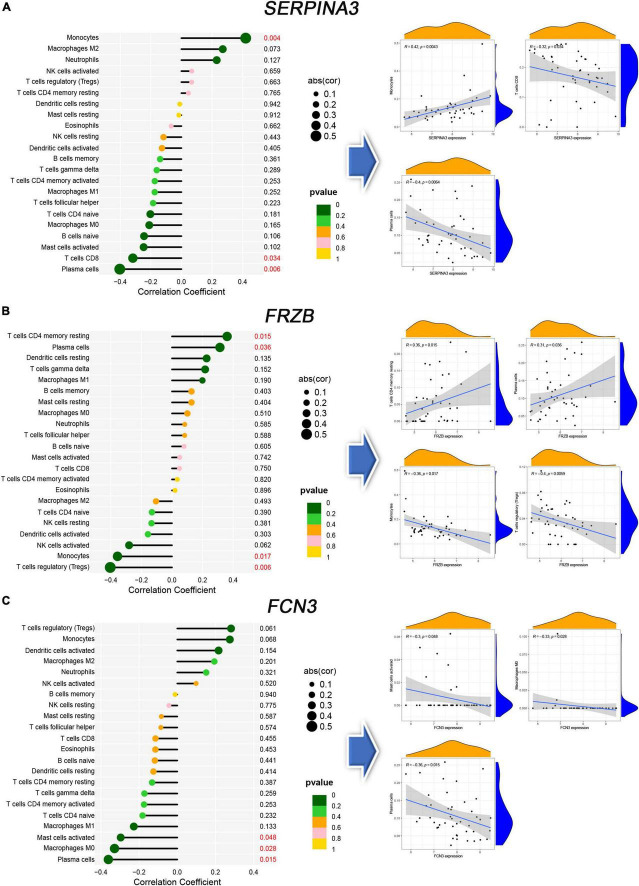
The immune correlation between diagnosis genes and immune cells. **(A–C)** The lollipop map and linear regression map in SERPINA3, FRAB, and FCN3.

The immune correlation between signal genes and 22 immune cells was applied to all 386 samples of six microarrays (Supplementary Figure 3). *SERPINA3* (Figure 7A) shows significant correlations in Monocytes, T cells CD8, and Plasma cells. Regarding *FRZB* (Figure 7B), the T cells CD4 memory resting, plasma cells, monocytes, and T cells regulatory (Tregs) show significant correlations. In *FCN3* (Figure 7C), the mast cells activated, macrophages M0, and plasma cells show significant correlations. These three genes show a typical significant immune cell, plasma cells. All three genes were correlated with plasma cells.

### 3.7. Differences in plasma proteins

**FIGURE 8:**
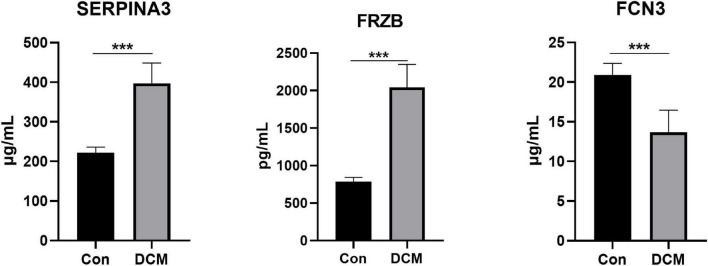
The plasma levels of SERPINA3, FRZB, and FCN3 in dilated cardiomyopathy (DCM) patients and healthy controls. ***Mean *P* < 0.001.

Finally, 24 individuals (12 healthy controls and 12 DCM patients) were recruited. We measured the plasma levels (Figure 8) of SERPINA3, FRZB, and FCN3. The plasma levels of SERPINA3 in DCM patients (397.17 ± 49.22 μg/ml) were higher (*P* < 0.001) than in healthy individuals (221.25 ± 14.15 μg/ml). Similarly, the plasma levels of FRZB in DCM patients (2,042.75 ± 292.62 pg/ml) were higher (*P* < 0.001) than in healthy individuals (784.58 ± 55.85 pg/ml). In FCN3, the plasma levels in DCM (13.67 ± 2.69 μg/ml) were lower than in the healthy control (20.92 ± 1.38 μg/ml). More importantly, all of the protein levels of these three genes were significant in DCMs compared with healthy controls.

## 4. Discussion

To our knowledge, this is the first work with normalized overall weights to filter candidate genes in DCM. Three genes, *SERPINA3*, *FRZB*, and *FCN3* show the AUC values in the training set (0.940, 0.889, and 0.887, respectively) and testing set (0.918, 0.911, and 0.901, respectively). In plasma protein, SERPINA3, FRZB, and FCN3 in DCM were significant compared with the control.

MLs have been extensively performed in four types of analysis, filtration of variables, classification, congression, and cluster. In bioinformatics, many studies take only one or two MLs, such as WGCNA (34), LASSO, and SVM. Nevertheless, a single ML might ignore the dominant variables. In our work (Table 1), the *FCN3* will be missed if just take the intersection of LASSO and SVM like the previous study (35). Various MLs showed their advantages. For instance, SVM and NN show their talents in the diagnosis of pigmented skin lesions (36). And in the pre-operative prediction of postsurgical mortality (37), GBM was the most MLs compared with DT, RF, and SVM. In our work, both RF and NN show their talent discrimination value in both training and testing sets with an accuracy of 100%. The normalized weights may be different even in the same variable (Table 1) in various MLs. So our work takes the sum of the normalized weights of different MLs into the following diagnosis value. Three tRNA, *SERPINA3*, *FRZB*, and *FCN3*, were filtered with a potential diagnosis of DCM. Furthermore, our method finds two potential diagnosis genes (FRZB and FCN3) in DCM that have never been reported before. Compared with previous studies, *SERPIAN3* presented the diagnosis value (38) in HF, and this work expanded its scale into DCM with the same point as Asakura and Kitakaze (39). Furthermore, Yang et al. (40) emphasizes the therapeutic value of *FRZB*, and our study expands its treatment potential to diagnosis value. Regarding *FCN3*, though studies pay attention to the diagnosis value for HF (41), no study reports the diagnosis value for DCM to our knowledge.

Serine proteinase inhibitor A3 (*SERPINA3*), also known as alpha-1 antichymotrypsin, has been shown to promote the development of cancer (42) and cardiac remodeling in patients with HF. In HF, though Delrue et al. (43) had confirmed that SERPINA3 is still an independent predictor of all-cause mortality, studies have paid little attention to the effect of pharmacological treatment of DCM. Spironolactone (44–47) dominates an important treated role in DCM. The previous study identified that spironolactone and lisinopril can downregulate SERPINA3 and treat mice with Duchenne muscular dystrophy, which suggests that SERPINA3 may be related to the salt corticosteroid receptor (48). Another study (49) came to a similar conclusion, *SERPINA3* was both upregulated *in vivo* (mice of mineralocorticoid receptor cardiac upregulation) and *in vitro* (H9C2 cells with aldosterone 24 h). The above studies indicated that the up-regulated of *SERPINA3* might be correlated with the mineralocorticoid receptor. However, few studies pay attention to DCM to our knowledge. And this work emphasizes the important role of *SERPINA3* in DCM.

*FRZB*, sFRP3 also named, is one of a frizzled-related proteins (FRPs) family (the other three were *sFRP-1*, *sFRP-2*, and *sFRP-4*). The *sFRP-3* and 4, can modulate apoptosis susceptibility in ventricular myocytes (50). However, though a previous study indicated that FRP contributed to the pathogenesis of DCM by down-regulated Wnt/β-catenin signaling pathway (51), no description of which of the four subtypes is associated. In DCM children (52), the serum circulating sFRP1 will trigger ventricular remodeling and cardiomyocyte fibrosis. And *sFRP-1* knockout mice (53) indicated an abnormal cardiac structure present with increasing age. And the *sFRP2* can prevent the conversion of inflammatory precursor components and the transformation of cardiomyocytes to pathogenic myofibroblasts (54) in DCM. However, no studies emphasized the function of FRZB in DCM, especially in plasma circulation. And our work first reported the diagnosis value of RZB in DCM. Furthermore, this work identified the significant upregulation of the circulation of FRZB protein in DCM.

*FCN3*, ficolin 3, was the most effective activator of the lectin pathway of complement (55) and more focus in rheumatic heart disease (56, 57). The *FCN3* is inversely associated with the severity of HF (58). Furthermore, lower *FCN3* is associated with the severity and outcome of HF (59). In congenital heart disease (60), the protein of FCN3 may prolong bleeding time and increase susceptibility to lung infection in the Fallot. However, few studies contribute to DCM. And our work first reported the diagnosis value of FCN3 in DCM. Furthermore, this work identified the significant downregulation of the circulation FCN3 protein in DCM.

Some limitations exist in our work. At first, inadequate validation is a common limitation in bioinformatics research. To decrease inadequate validation, three methods were taken, increase the sample size, developed the testing sets, and add little sample size clinical validation. However, additional studies should be conducted to validate, including but not limited to large sample size clinical trials or animal experiments for reliable verification of our predicted results. Secondly, MLs models exists some inevitable limitations, such as black box phenomenon (61), especially in NN (62) which contains various layers (e.g., an input layer, an output layer, and several hidden layers). Finally, few clinical features can be obtained, such as the age (63) or race (64) of the patient, which might trigger the bias of the result. In summary, further subgroup analyses are expected to assess more valuable conclusions in future works.

## 5. Conclusion

The overall weights methods for the filtration of genes in six MLs were developed, and we successfully found validation of three diagnosis genes, *SERPINA3*, *FRZB*, and *FCN3*. Further verification work could be implemented.

## Data availability statement

The datasets presented in this study can be found in online repositories. The names of the repository/repositories and accession number(s) can be found below: https://www.ncbi.nlm.nih.gov/geo/query/acc.cgi?acc=GSE5406; https://www.ncbi.nlm.nih.gov/geo/query/acc.cgi?acc=GSE57338; https://www.ncbi.nlm.nih.gov/geo/query/acc.cgi?acc=GSE1145; https://www.ncbi.nlm.nih.gov/geo/query/acc.cgi?acc=GSE1869; https://www.ncbi. nlm.nih.gov/geo/query/acc.cgi?acc=GSE3585; and https://www.ncbi.nlm.nih.gov/geo/query/acc.cgi?acc=GSE42955.

## Ethics statement

The studies involving human participants were reviewed and approved by Ethics Review Committee Jinghai District Hospital. The patients/participants provided their written informed consent to participate in this study.

## Author contributions

LZ and YL: conceptualization, investigation, and data curation. LZ and KW: methodology. LZ: software and writing—original draft preparation. YL, KW, LZ, LH, HY, XF, XG, and JZ: validation. KW: writing—review and editing. ZL, HY, and XF: supervision. HZ and LH: project administration. HY: funding acquisition. All authors contributed to the article and approved the submitted version.
