# Supplementary material for: Multiple-model machine learning identifies potential functional genes in dilated cardiomyopathy
Source: Front Cardiovasc Med. 2023 Jan 11;9:1044443. doi: 10.3389/fcvm.2022.1044443 (PMC9874116; doi:10.3389/fcvm.2022.1044443)

**Multiple-Model Machine Learning Identifies Potential Functional Genes in Dilated Cardiomyopathy**

**Content**

Table S1. The 20 DEGs in healthy and DCM.

Table S2. GSEA enrichment of 21terms.

Table S3. GO enrichment of 102 terms.

Table S4. DO enrichment of 41 terms.

Table S5. Primary weight of DEGs in the six classifications MLs.

Fig.S1 ROC curve in the training group.

Fig.S2 ROC curve in the testing group.

Fig.S3 The Correlation map between immune cells.

Table S1. The 20 DEGs in healthy and DCM.

| Genes | logFC | AveExpr | t | P.Value | adj.P.Val | B |
| --- | --- | --- | --- | --- | --- | --- |
| SERPINA3 | -1.99 | 7.4 | -20.12 | 6.01E-59 | 7.78E-55 | 123.19 |
| ASPN | 1.67 | 7.68 | 15.68 | 1.38E-41 | 4.37E-38 | 83.75 |
| SLCO4A1 | -1.07 | 6.81 | -15.65 | 1.69E-41 | 4.37E-38 | 83.55 |
| CD163 | -1.38 | 7.79 | -15.5 | 6.68E-41 | 1.44E-37 | 82.19 |
| FRZB | 1.1 | 5.87 | 15.48 | 8.34E-41 | 1.54E-37 | 81.97 |
| LUM | 1.19 | 8.92 | 15.13 | 1.75E-39 | 2.26E-36 | 78.97 |
| SFRP4 | 1.22 | 5.29 | 15.07 | 3.12E-39 | 3.67E-36 | 78.39 |
| LYVE1 | -1.13 | 8.56 | -14.69 | 9.27E-38 | 7.06E-35 | 75.04 |
| FCN3 | -1.2 | 7.91 | -14.36 | 1.60E-36 | 1.09E-33 | 72.23 |
| IL1RL1 | -1.41 | 5.59 | -13.95 | 5.91E-35 | 3.64E-32 | 68.67 |
| VSIG4 | -1.01 | 7.49 | -13.5 | 2.98E-33 | 1.43E-30 | 64.8 |
| PLA2G2A | -1.54 | 8.16 | -12.83 | 9.69E-31 | 3.14E-28 | 59.09 |
| MXRA5 | 1.04 | 7.41 | 11.77 | 7.30E-27 | 1.13E-24 | 50.28 |
| CYP4B1 | -1.16 | 6.6 | -11.26 | 4.93E-25 | 5.46E-23 | 46.12 |
| ANKRD2 | -1.01 | 9.16 | -11.22 | 6.93E-25 | 7.20E-23 | 45.79 |
| MYOT | -1.03 | 6.96 | -11.04 | 3.02E-24 | 2.81E-22 | 44.33 |
| SERPINE1 | -1.34 | 7.11 | -10.02 | 9.14E-21 | 4.79E-19 | 36.44 |
| NPPA | 1.66 | 8.67 | 9 | 1.94E-17 | 5.29E-16 | 28.9 |
| S100A8 | -1.08 | 7.68 | -8.73 | 1.41E-16 | 3.32E-15 | 26.95 |
| HBB | 1.13 | 8.47 | 7.08 | 9.00E-12 | 9.26E-11 | 16.11 |

* logFC, the expression value of of fold change; AveExpr, the average expression of genes; adj.P.Val, the adjusted value of P.

Table S2. GSEA enrichment of 21terms.

| ID | setSize | enrichmentScore | NES | pvalue | p.adjust |
| --- | --- | --- | --- | --- | --- |
| Type I Diabetes Mellitus | 37 | 0.72 | 2.24 | 1.12879E-06 | 0.000206569 |
| Graft Versus Host Disease | 33 | 0.69 | 2.07 | 2.80189E-05 | 0.001631139 |
| Allograft Rejection | 32 | 0.68 | 2.04 | 4.104E-05 | 0.001631139 |
| Pathogenic Escherichia Coli Infection | 49 | -0.61 | -1.95 | 4.45666E-05 | 0.001631139 |
| Parkinsons Disease | 88 | 0.53 | 1.9 | 1.86379E-05 | 0.001631139 |
| Antigen Processing And Presentation | 70 | 0.55 | 1.9 | 5.54906E-05 | 0.001692462 |
| Hematopoietic Cell Lineage | 84 | -0.52 | -1.8 | 0.000276926 | 0.007088305 |
| Vegf Signaling Pathway | 70 | -0.52 | -1.75 | 0.000309871 | 0.007088305 |
| Complement And Coagulation Cascades | 67 | -0.53 | -1.77 | 0.000357124 | 0.007261526 |
| Acute Myeloid Leukemia | 57 | -0.53 | -1.73 | 0.001061838 | 0.016852483 |
| Glycerophospholipid Metabolism | 64 | -0.51 | -1.71 | 0.001127219 | 0.016852483 |
| Oxidative Phosphorylation | 87 | 0.46 | 1.66 | 0.001197171 | 0.016852483 |
| Mapk Signaling Pathway | 247 | -0.37 | -1.5 | 0.000989544 | 0.016852483 |
| Autoimmune Thyroid Disease | 42 | 0.59 | 1.86 | 0.001550475 | 0.020266922 |
| Intestinal Immune Network For Iga Production | 41 | 0.55 | 1.73 | 0.00218262 | 0.026627961 |
| Fc Gamma R Mediated Phagocytosis | 90 | -0.45 | -1.58 | 0.00243152 | 0.02781051 |
| Apoptosis | 84 | -0.47 | -1.64 | 0.002890776 | 0.030870014 |
| B Cell Receptor Signaling Pathway | 71 | -0.48 | -1.62 | 0.003036395 | 0.030870014 |
| Chronic Myeloid Leukemia | 72 | -0.48 | -1.64 | 0.004784295 | 0.045566776 |
| P53 Signaling Pathway | 62 | -0.48 | -1.59 | 0.005050258 | 0.045566776 |
| Peroxisome | 71 | 0.45 | 1.56 | 0.005228974 | 0.045566776 |

Table S3. GO enrichment of 102 terms.

| ONTOLOGY | Description | GeneRatio | pvalue | p.adjust |
| --- | --- | --- | --- | --- |
| BP | Cellular Zinc Ion Homeostasis | 4/65 | 9.77E-06 | 0.004176 |
| BP | Positive Regulation Of Inflammatory Response | 6/65 | 1.08E-05 | 0.004176 |
| BP | Zinc Ion Homeostasis | 4/65 | 1.20E-05 | 0.004176 |
| BP | Detoxification | 6/65 | 1.31E-05 | 0.004176 |
| BP | Neutrophil Chemotaxis | 5/65 | 3.27E-05 | 0.008355 |
| BP | Collagen Fibril Organization | 4/65 | 6.24E-05 | 0.011038 |
| BP | Granulocyte Chemotaxis | 5/65 | 8.06E-05 | 0.011038 |
| BP | Neutrophil Migration | 5/65 | 8.06E-05 | 0.011038 |
| BP | Extracellular Matrix Organization | 7/65 | 8.95E-05 | 0.011038 |
| BP | Extracellular Structure Organization | 7/65 | 9.13E-05 | 0.011038 |
| BP | External Encapsulating Structure Organization | 7/65 | 9.51E-05 | 0.011038 |
| BP | Positive Regulation Of Phagocytosis | 4/65 | 0.000106 | 0.011307 |
| BP | Myeloid Leukocyte Migration | 6/65 | 0.000139 | 0.013626 |
| BP | Leukocyte Chemotaxis | 6/65 | 0.000163 | 0.014905 |
| BP | Granulocyte Migration | 5/65 | 0.000192 | 0.016356 |
| BP | Response To Toxic Substance | 6/65 | 0.000209 | 0.016691 |
| BP | Cardiac Muscle Hypertrophy | 4/65 | 0.000341 | 0.023654 |
| BP | Leukocyte Migration | 7/65 | 0.000353 | 0.023654 |
| BP | Striated Muscle Hypertrophy | 4/65 | 0.000383 | 0.023654 |
| BP | Regulation Of Phagocytosis | 4/65 | 0.000383 | 0.023654 |
| BP | Cellular Oxidant Detoxification | 4/65 | 0.000398 | 0.023654 |
| BP | Muscle Hypertrophy | 4/65 | 0.000413 | 0.023654 |
| BP | Regulation Of Blood Pressure | 5/65 | 0.000426 | 0.023654 |
| BP | Positive Regulation Of Defense Response | 6/65 | 0.000484 | 0.025735 |
| BP | Thyroid Hormone Transport | 2/65 | 0.00052 | 0.026577 |
| BP | Acute Inflammatory Response | 4/65 | 0.000632 | 0.030775 |
| BP | Cellular Transition Metal Ion Homeostasis | 4/65 | 0.000675 | 0.030775 |
| BP | Cellular Detoxification | 4/65 | 0.000675 | 0.030775 |
| BP | Muscle Adaptation | 4/65 | 0.000743 | 0.032336 |
| BP | Cell Chemotaxis | 6/65 | 0.00076 | 0.032336 |
| BP | Positive Regulation Of Response To External Stimulus | 7/65 | 0.000814 | 0.032595 |
| BP | Response To Zinc Ion | 3/65 | 0.000817 | 0.032595 |
| BP | Cellular Response To Toxic Substance | 4/65 | 0.000868 | 0.032685 |
| BP | Muscle System Process | 7/65 | 0.000892 | 0.032685 |
| BP | Leukocyte Aggregation | 2/65 | 0.000896 | 0.032685 |
| BP | Detoxification Of Copper Ion | 2/65 | 0.001201 | 0.039732 |
| BP | Oxygen Transport | 2/65 | 0.001201 | 0.039732 |
| BP | Stress Response To Copper Ion | 2/65 | 0.001201 | 0.039732 |
| BP | Cardiac Muscle Tissue Development | 5/65 | 0.001288 | 0.039732 |
| BP | Transition Metal Ion Homeostasis | 4/65 | 0.001366 | 0.039732 |
| BP | Opsonization | 2/65 | 0.001369 | 0.039732 |
| BP | Protein Nitrosylation | 2/65 | 0.001369 | 0.039732 |
| BP | Peptidyl-Cysteine S-Nitrosylation | 2/65 | 0.001369 | 0.039732 |
| BP | Sequestering Of Metal Ion | 2/65 | 0.001369 | 0.039732 |
| BP | Response To Fungus | 3/65 | 0.001414 | 0.040123 |
| BP | Chronic Inflammatory Response | 2/65 | 0.001548 | 0.041964 |
| BP | Replicative Senescence | 2/65 | 0.001548 | 0.041964 |
| BP | Negative Regulation Of Growth | 5/65 | 0.001577 | 0.041964 |
| BP | Striated Muscle Tissue Development | 5/65 | 0.001664 | 0.043364 |
| BP | Detoxification Of Inorganic Compound | 2/65 | 0.001738 | 0.044381 |
| BP | Leukocyte Migration Involved In Inflammatory Response | 2/65 | 0.001938 | 0.047589 |
| BP | Stress Response To Metal Ion | 2/65 | 0.001938 | 0.047589 |
| BP | Negative Regulation Of Smooth Muscle Cell Proliferation | 3/65 | 0.002065 | 0.049743 |
| BP | Gas Transport | 2/65 | 0.002369 | 0.055188 |
| BP | Regulation Of Inflammatory Response | 6/65 | 0.002377 | 0.055188 |
| BP | Negative Regulation Of Viral Entry Into Host Cell | 2/65 | 0.0026 | 0.059297 |
| CC | Collagen-Containing Extracellular Matrix | 21/65 | 2.16E-19 | 2.34E-17 |
| CC | Vacuolar Lumen | 6/65 | 2.41E-05 | 0.0013 |
| CC | Lysosomal Lumen | 4/65 | 0.000303 | 0.008047 |
| CC | Golgi Lumen | 4/65 | 0.000395 | 0.008047 |
| CC | Interstitial Matrix | 2/65 | 0.000585 | 0.008047 |
| CC | Haptoglobin-Hemoglobin Complex | 2/65 | 0.000585 | 0.008047 |
| CC | Secretory Granule Lumen | 6/65 | 0.000688 | 0.008047 |
| CC | Hemoglobin Complex | 2/65 | 0.0007 | 0.008047 |
| CC | Cytoplasmic Vesicle Lumen | 6/65 | 0.000722 | 0.008047 |
| CC | Vesicle Lumen | 6/65 | 0.000745 | 0.008047 |
| CC | I Band | 4/65 | 0.001174 | 0.01153 |
| CC | Blood Microparticle | 4/65 | 0.001444 | 0.012993 |
| CC | Endocytic Vesicle Lumen | 2/65 | 0.002621 | 0.021774 |
| CC | Collagen Trimer | 3/65 | 0.002931 | 0.022607 |
| CC | Sarcomere | 4/65 | 0.005126 | 0.036905 |
| CC | Myofibril | 4/65 | 0.006945 | 0.046881 |
| CC | Contractile Fiber | 4/65 | 0.008055 | 0.051175 |
| CC | Z Disc | 3/65 | 0.008671 | 0.052024 |
| MF | Extracellular Matrix Structural Constituent | 10/64 | 3.82E-10 | 6.72E-08 |
| MF | Extracellular Matrix Structural Constituent Conferring Compression Resistance | 5/64 | 1.09E-08 | 9.59E-07 |
| MF | Long-Chain Fatty Acid Binding | 3/64 | 1.77E-05 | 0.001038 |
| MF | Collagen Binding | 4/64 | 9.15E-05 | 0.004027 |
| MF | Organic Acid Binding | 5/64 | 0.00013 | 0.004582 |
| MF | Antioxidant Activity | 4/64 | 0.000218 | 0.006387 |
| MF | Oxygen Binding | 3/64 | 0.000335 | 0.00842 |
| MF | Structural Constituent Of Muscle | 3/64 | 0.000418 | 0.009186 |
| MF | Haptoglobin Binding | 2/64 | 0.000526 | 0.009255 |
| MF | Rage Receptor Binding | 2/64 | 0.000526 | 0.009255 |
| MF | Fatty Acid Binding | 3/64 | 0.000659 | 0.010538 |
| MF | Toll-Like Receptor Binding | 2/64 | 0.000768 | 0.010617 |
| MF | Peroxidase Activity | 3/64 | 0.000784 | 0.010617 |
| MF | Oxidoreductase Activity, Acting On Peroxide As Acceptor | 3/64 | 0.000974 | 0.012243 |
| MF | Oxygen Carrier Activity | 2/64 | 0.001054 | 0.012365 |
| MF | Glycosaminoglycan Binding | 5/64 | 0.001317 | 0.014323 |
| MF | Opsonin Binding | 2/64 | 0.001383 | 0.014323 |
| MF | Complement Binding | 2/64 | 0.002171 | 0.021227 |
| MF | Monocarboxylic Acid Binding | 3/64 | 0.002818 | 0.026106 |
| MF | Hyaluronic Acid Binding | 2/64 | 0.003126 | 0.027505 |
| MF | Endopeptidase Inhibitor Activity | 4/64 | 0.003557 | 0.029811 |
| MF | Peptidase Inhibitor Activity | 4/64 | 0.004074 | 0.032596 |
| MF | Endopeptidase Regulator Activity | 4/64 | 0.004641 | 0.034218 |
| MF | Serine-Type Endopeptidase Inhibitor Activity | 3/64 | 0.004818 | 0.034218 |
| MF | Wnt-Protein Binding | 2/64 | 0.00486 | 0.034218 |
| MF | Receptor Ligand Activity | 6/64 | 0.006953 | 0.047065 |
| MF | Signaling Receptor Activator Activity | 6/64 | 0.007434 | 0.048461 |
| MF | Peptidase Regulator Activity | 4/64 | 0.008399 | 0.052791 |

* MF:molecular function; BP: biological process; CC: cellular components

Table S4. DO enrichment of 41 terms.

| Description | GeneRatio | BgRatio | pvalue | p.adjust |
| --- | --- | --- | --- | --- |
| Atherosclerosis | 12/49 | 344/8007 | 7.16E-07 | 0.000134 |
| Arteriosclerotic Cardiovascular Disease | 12/49 | 345/8007 | 7.39E-07 | 0.000134 |
| Arteriosclerosis | 12/49 | 356/8007 | 1.03E-06 | 0.000134 |
| Extrinsic Cardiomyopathy | 4/49 | 26/8007 | 1.68E-05 | 0.001311 |
| Myocarditis | 4/49 | 26/8007 | 1.68E-05 | 0.001311 |
| Psoriatic Arthritis | 3/49 | 10/8007 | 2.51E-05 | 0.001634 |
| Lung Disease | 12/49 | 499/8007 | 3.32E-05 | 0.001854 |
| Kidney Failure | 7/49 | 156/8007 | 4.02E-05 | 0.001967 |
| Myopathy | 10/49 | 398/8007 | 0.000118 | 0.004619 |
| Muscle Tissue Disease | 10/49 | 398/8007 | 0.000118 | 0.004619 |
| Muscular Disease | 10/49 | 408/8007 | 0.000145 | 0.005155 |
| Coronary Stenosis | 3/49 | 18/8007 | 0.000165 | 0.005369 |
| Prostate Cancer | 10/49 | 425/8007 | 0.000203 | 0.00588 |
| Coronary Artery Disease | 9/49 | 348/8007 | 0.000219 | 0.00588 |
| Esophageal Cancer | 6/49 | 145/8007 | 0.000234 | 0.00588 |
| Male Reproductive Organ Cancer | 10/49 | 435/8007 | 0.000245 | 0.00588 |
| Myocardial Infarction | 8/49 | 279/8007 | 0.000256 | 0.00588 |
| Cardiomyopathy | 6/49 | 149/8007 | 0.000271 | 0.005895 |
| Type 2 Diabetes Mellitus | 7/49 | 215/8007 | 0.0003 | 0.006183 |
| Obstructive Lung Disease | 8/49 | 308/8007 | 0.000498 | 0.009734 |
| Esophagus Squamous Cell Carcinoma | 4/49 | 63/8007 | 0.000566 | 0.010531 |
| Breast Ductal Carcinoma | 3/49 | 29/8007 | 0.000704 | 0.012507 |
| Esophageal Carcinoma | 5/49 | 123/8007 | 0.000876 | 0.014267 |
| Pulmonary Fibrosis | 5/49 | 123/8007 | 0.000876 | 0.014267 |
| Glomerulonephritis | 4/49 | 79/8007 | 0.001328 | 0.02077 |
| Familial Hyperlipidemia | 4/49 | 82/8007 | 0.001525 | 0.022938 |
| Sarcoidosis | 4/49 | 84/8007 | 0.001668 | 0.023427 |
| Kidney Disease | 9/49 | 461/8007 | 0.001678 | 0.023427 |
| Nephritis | 5/49 | 147/8007 | 0.001939 | 0.02497 |
| Exostosis | 2/49 | 11/8007 | 0.001948 | 0.02497 |
| Hypersensitivity Reaction Type Iv Disease | 4/49 | 88/8007 | 0.00198 | 0.02497 |
| Chronic Obstructive Pulmonary Disease | 6/49 | 220/8007 | 0.002094 | 0.025523 |
| Urinary System Disease | 9/49 | 478/8007 | 0.002154 | 0.025523 |
| Bronchial Disease | 5/49 | 153/8007 | 0.002311 | 0.026031 |
| Lipid Metabolism Disorder | 4/49 | 92/8007 | 0.00233 | 0.026031 |
| Fatty Liver Disease | 4/49 | 95/8007 | 0.002619 | 0.02845 |
| Keratoacanthoma | 2/49 | 14/8007 | 0.003186 | 0.033653 |
| Lymphatic System Disease | 4/49 | 101/8007 | 0.003271 | 0.033653 |
| Interstitial Lung Disease | 5/49 | 170/8007 | 0.003646 | 0.036406 |
| Inherited Metabolic Disorder | 7/49 | 331/8007 | 0.003724 | 0.036406 |
| Pneumonia | 4/49 | 108/8007 | 0.004161 | 0.03968 |
| Kawasaki Disease | 3/49 | 54/8007 | 0.004291 | 0.039951 |
| Respiratory Failure | 3/49 | 55/8007 | 0.00452 | 0.040886 |
| Membranous Glomerulonephritis | 2/49 | 17/8007 | 0.004706 | 0.040886 |
| Atrial Heart Septal Defect | 2/49 | 17/8007 | 0.004706 | 0.040886 |
| Lymphadenitis | 3/49 | 58/8007 | 0.005248 | 0.041877 |
| Retinal Vascular Disease | 3/49 | 58/8007 | 0.005248 | 0.041877 |
| Diabetic Retinopathy | 3/49 | 58/8007 | 0.005248 | 0.041877 |
| Lymph Node Disease | 3/49 | 58/8007 | 0.005248 | 0.041877 |
| Retinal Disease | 7/49 | 358/8007 | 0.005717 | 0.044143 |
| Pulmonary Tuberculosis | 2/49 | 19/8007 | 0.005871 | 0.044143 |
| Iga Glomerulonephritis | 2/49 | 19/8007 | 0.005871 | 0.044143 |
| Periodontitis | 4/49 | 120/8007 | 0.006046 | 0.0446 |
| Sarcoma | 5/49 | 193/8007 | 0.006236 | 0.044733 |
| Hypertrophic Cardiomyopathy | 3/49 | 62/8007 | 0.006324 | 0.044733 |
| Prostate Carcinoma | 4/49 | 122/8007 | 0.006407 | 0.044733 |
| Lipid Storage Disease | 4/49 | 130/8007 | 0.007995 | 0.054846 |
| Chronic Kidney Failure | 3/49 | 70/8007 | 0.008845 | 0.056773 |
| Asthma | 4/49 | 134/8007 | 0.008879 | 0.056773 |
| Intrinsic Cardiomyopathy | 4/49 | 135/8007 | 0.00911 | 0.056773 |
| Heart Septal Defect | 2/49 | 24/8007 | 0.009293 | 0.056773 |
| Skin Squamous Cell Carcinoma | 2/49 | 24/8007 | 0.009293 | 0.056773 |
| Acne | 2/49 | 24/8007 | 0.009293 | 0.056773 |
| Sebaceous Gland Disease | 2/49 | 24/8007 | 0.009293 | 0.056773 |
| Familial Hypercholesterolemia | 2/49 | 25/8007 | 0.010062 | 0.059661 |
| Periodontal Disease | 4/49 | 139/8007 | 0.010071 | 0.059661 |
| Hyperostosis | 2/49 | 26/8007 | 0.010858 | 0.062557 |
| Chronic Lymphocytic Leukemia | 5/49 | 221/8007 | 0.01088 | 0.062557 |

Table S5. Primary weight of DEGs in the six classifications MLs.

| Genes | LASSO | RF | NN | GBM | DT | SVM |
| --- | --- | --- | --- | --- | --- | --- |
| SERPINA3 | -0.99 | 30.72 | -1 | 1624.19 | 107.77 | 28.19 |
| CD163 | 0 | 11.39 | -1.37 | 314.02 | 86.91 | 2.33 |
| FCN3 | 0 | 9.94 | -1.24 | 14.09 | 78.57 | 2.39 |
| LYVE1 | -0.07 | 12.61 | 0.7 | 55.03 | 77.87 | 4.51 |
| SLCO4A1 | 0 | 12.54 | 0.3 | 219.32 | 82.74 | 2.32 |
| LUM | 0.31 | 8.68 | -0.9 | 32.77 | 0 | 1.96 |
| FRZB | 0.17 | 10.79 | -0.34 | 138.83 | 0 | 7.59 |
| PLA2G2A | 0 | 6.39 | 0.14 | 26.71 | 78.57 | 1.13 |
| SFRP4 | 0 | 4.56 | 1.14 | 29.66 | 0 | 1.64 |
| NPPA | 0.1 | 5.41 | -0.49 | 90.51 | 0 | 2.89 |
| MYOT | 0 | 3.38 | 0.88 | 9.95 | 0 | 0.76 |
| ASPN | 0.09 | 8.4 | 0.37 | 34.85 | 0 | 2.38 |
| ANKRD2 | -0.27 | 5.39 | -0.12 | 119.66 | 0 | 2.73 |
| MXRA5 | 0 | 2.29 | -0.63 | 7.07 | 0 | 0.5 |
| HBB | 0.09 | 3.27 | 0.41 | 31.16 | 0 | 0.83 |
| IL1RL1 | 0 | 9 | -0.11 | 19.36 | 0 | 2.11 |
| S100A8 | 0 | 2.19 | -0.47 | 5.04 | 0 | 1.17 |
| CYP4B1 | -0.06 | 4.83 | -0.15 | 31.23 | 0 | 1.31 |
| VSIG4 | 0 | 4.99 | 0.06 | 6.63 | 0 | 0.62 |
| SERPINE1 | 0 | 2.37 | 0.1 | 7.87 | 0 | 0.54 |

*LASSO, Least Absolute Shrinkage and Selection Operator; RF, Random Forest; GBM, Gradient Boosting Machine; DT, Decision Trees ; NN, Neural Network.

Fig.S1 ROC curve in the training group.


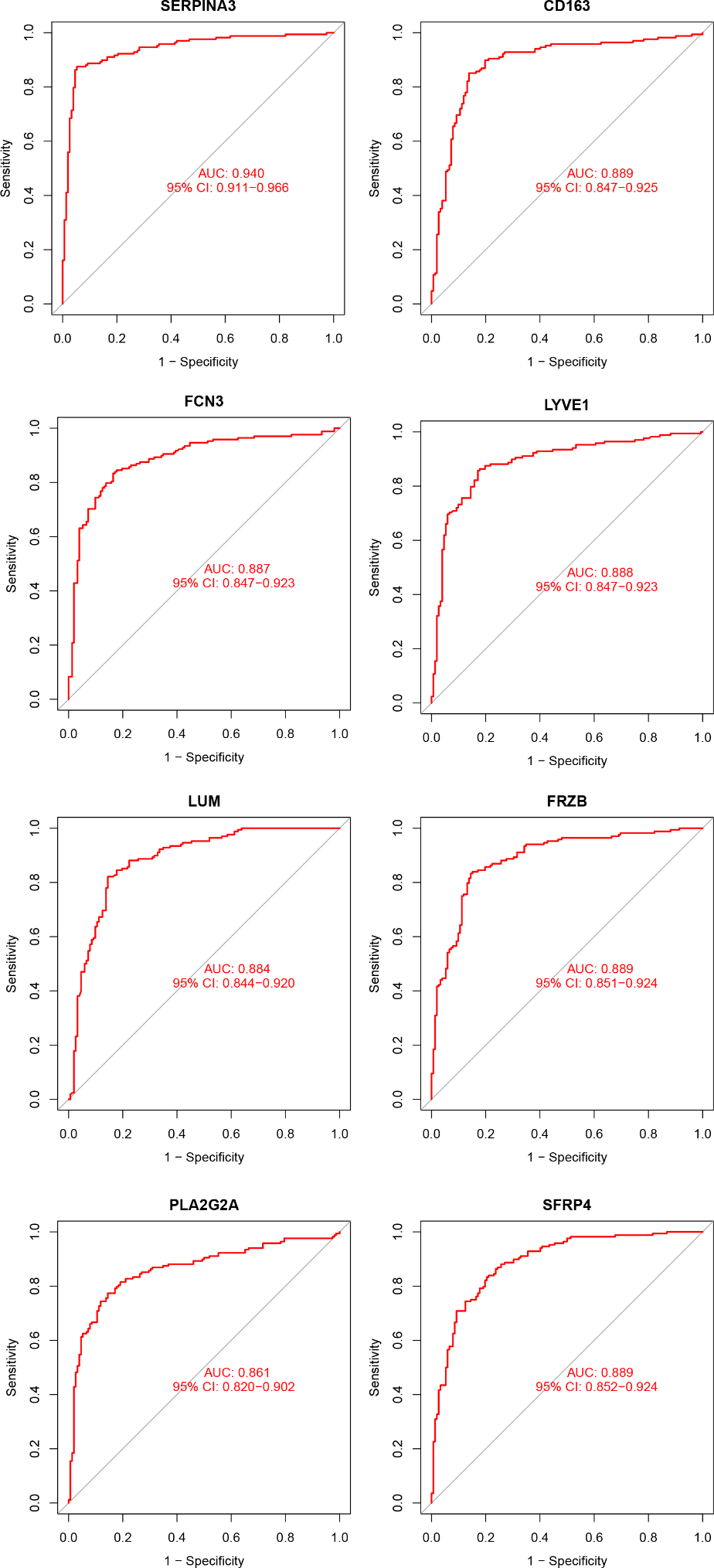


Fig.S2 ROC curve in the testing group.


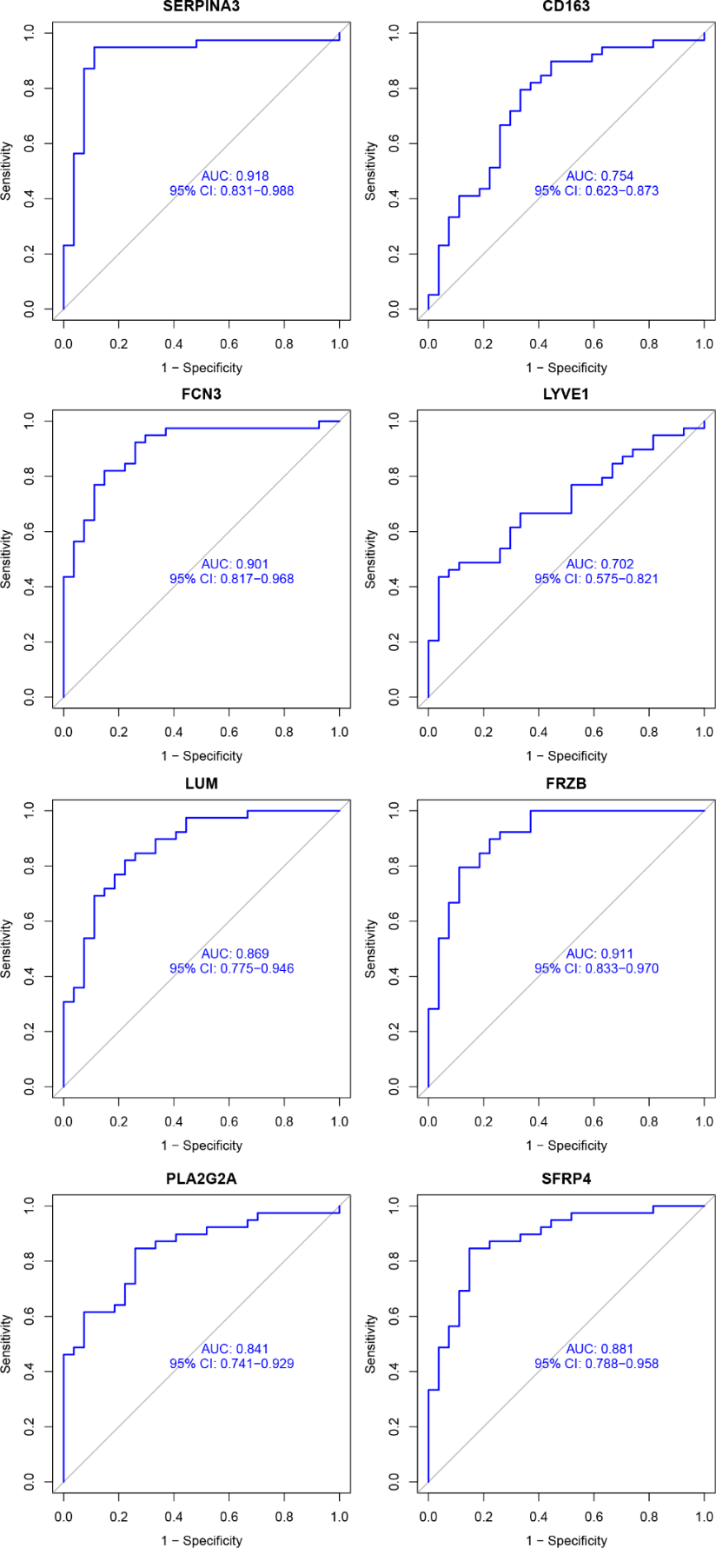


Fig.S3 The Correlation map between immune cells.


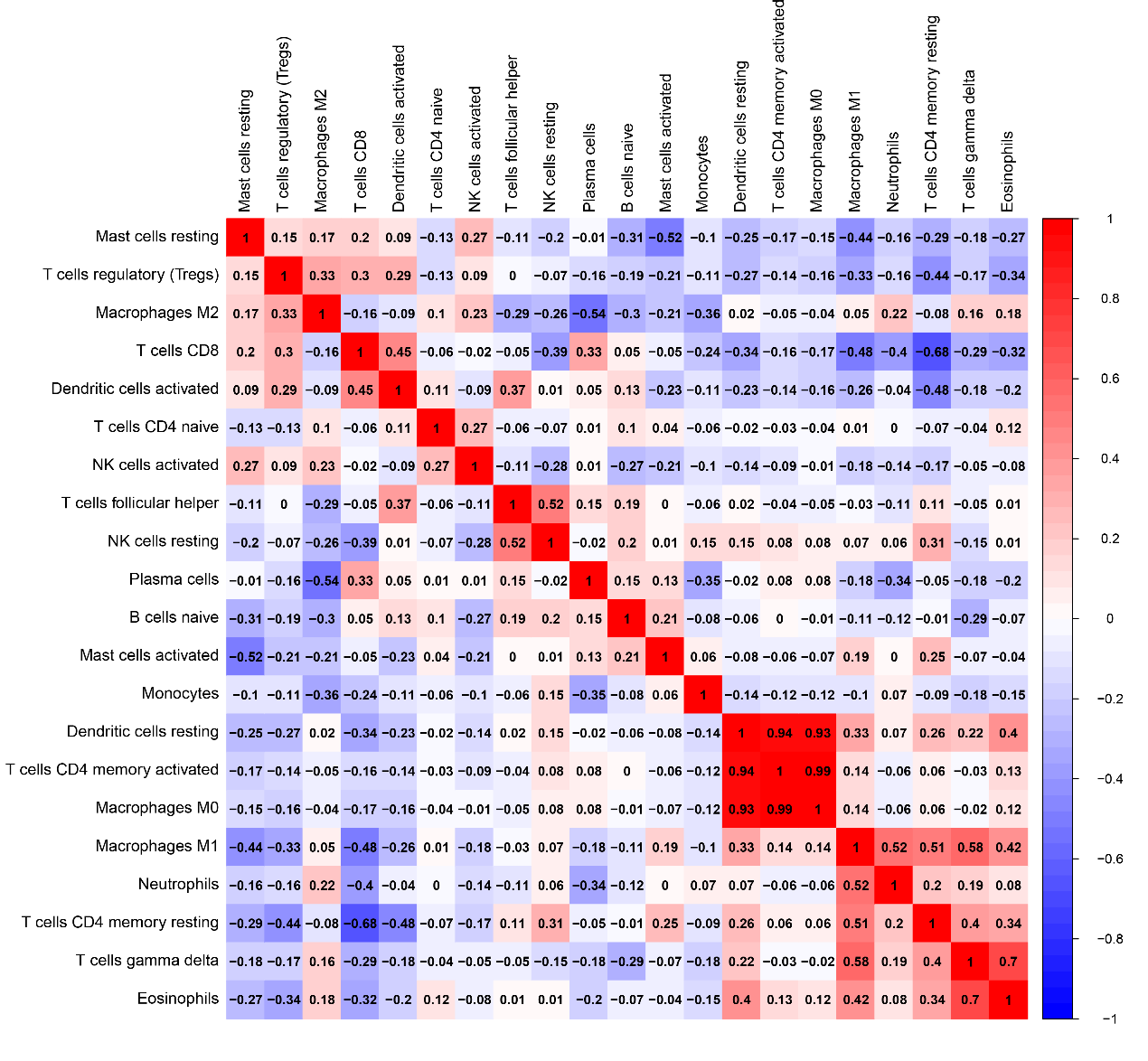

Supplement: Supplementary file 1 [file Data_Sheet_1.docx]
